# 3D Printed Double Roller-Based Triboelectric Nanogenerator for Blue Energy Harvesting

**DOI:** 10.3390/mi12091089

**Published:** 2021-09-10

**Authors:** Inkyum Kim, Daewon Kim

**Affiliations:** 1 Department of Electronics and Information Convergence Engineering, Kyung Hee University, 1732 Deogyeong-daero, Giheung-gu, Yongin 17104, Korea; inkyum.kim@khu.ac.kr; 2 Institute for Wearable Convergence Electronics, Kyung Hee University, 1732 Deogyeong-daero, Giheung-gu, Yongin 17104, Korea; 3 Department of Electronic Engineering, Kyung Hee University, 1732 Deogyeong-daero, Giheung-gu, Yongin 17104, Korea

**Keywords:** triboelectric nanogenerators, blue energy harvesting, double rollers, space-conservative method, ecologically friendly technology

## Abstract

The ocean covers 70% of the earth’s surface and is one of the largest uncultivated resources still available for harvesting energy. The triboelectric energy harvesting technology has the potential to effectively convert the ocean’s “blue energy” into electricity. A half-cylinder structure including rollers floating on the water has already been used, in which the pendulum motion of the rollers is driven by the waveform. For the stable motion of the rollers, the printed surface of the device was treated with acetone for attaining hydrophilicity. The electrical outputs with the proposed device were enhanced by increasing the contact surface area by simply implementing the double roller structure with double side-covered electrodes. With the optimized structure, the maximum power density reached a value of 69.34 µW m^−2^ at a load resistance of 200 MΩ with the device’s high output durability. Finally, the fabricated device was also applied to the artificial water waves to demonstrate the possibility of using this device in the ocean. By simply modifying the electrode structure and adding a roller, this device demonstrated the ability to generate over 160% of electrical output with the same covered area of the ocean by the triboelectric nanogenerators (TENGs) and potential ocean application.

## 1. Introduction

The ocean, which covers 70% of earth’s surface area, offers a potentially infinite source of renewable energy in the form of waves [1]. The wave energy in the ocean occurs and disappears in the form of potential energy. When this wasted wave energy is effectively harvested, the growing demand for energy could be allocated, and the problem of fossil fuel depletion will be mitigated simultaneously [2,3]. Energy harvesting technology, which collects energy from an ambient environment, can be applied to scavenge the water wave energy and generate electricity. Energy harvesting is a process of deriving, capturing, and storing energy from external sources with small electronic devices [4]. As a demonstration of the ability to harvest energy from the water wave energy, a large-scale harvesting device has already been represented to build structures on the ocean [5,6,7].

Among the various types of energy harvesting devices, electromagnetic generators (EMG) and triboelectric nanogenerators (TENG) have been predominantly used to capture energy from water waves by exploiting wave energy, based on their individual working principles. EMG devices consist of coils and magnets and generate electricity from a changing magnetic flux induced through the coil by a moving magnet, following Faraday’s law [8]. EMGs can generate electricity from spinning turbines in tidal currents or magnets oscillated by the water’s kinetic energy [9]. EMGs typically exhibit high current output because of their low internal resistance and the relatively heavy weight from the mass of the magnets. A TENG operates using simple contact-separation between two materials, based on the principle of contact electrification and electrostatic induction [10,11,12]. The device consists of two different contact materials and one or more electrodes to induce charge flow [13,14,15]. TENGs exhibit high output power, light weight, and a simple device structure [16,17,18]. Due to the wide range of materials selection, TENGs can also be used as wearable sensors or skin attachable devices [19,20,21,22]. By employing robust materials from the broad material choices, TENGs also can generate electricity in relatively harsh conditions [23,24,25].

Diverse types of blue energy harvesters, including a film type TENG installed at the shore, a pendulum-shaped oscillator, and contact-separation with zigzag structures, have already been reported [26,27,28,29,30,31]. For water energy harvesting, one of the effective designs is a freestanding TENG with a freely moving component between two electrodes, which can exploit the input kinetic energy from the lateral movement of waves [32,33,34,35,36]. Rollers were previously employed on these freestanding TENG devices with a one-sided electrode TENG and on a sphere type TENG [37,38,39,40,41]. The TENG with rollers represented advantages in unbounded movable space for the rollers with effectively transferred input force. Moreover, with the cylindrical roller, one TENG device can harvest the bidirectional wave energy. However, there is a new method for enhancing electrical outputs with the same size as the roller-based TENG device.

In this study, a double roller-based TENG (DR-TENG) is introduced to increase the degree of tribo-charge accumulation on the contact electrode surface while occupying the same volume. Double side-covered electrodes can also be adopted to increase the total effective contact area between rollers and electrodes and the electrical outputs. By applying this concept of the double side-covered electrodes and double rollers to the conventional roller based-TENG, the electrical output can be enhanced as a space conservative method. For easy implementation of the DR structure to a TENG device, three-dimensional (3D) printing technology can be used to fabricate the frame and various sizes of the roller while presenting the reproducible property with the same drawing. The fitted size of aluminium (Al) tape is cut and attached to the printed structure. A hydrophilic treatment for the 3D printed surface is conducted for the stable movement of the rollers in water wave conditions. To manifest the operation of the DR-TENG, charge distribution and current flow mechanism are analyzed, and a finite element method (FEM) simulation is conducted. After structural optimization, the electrical outputs of open-circuit voltage (*V*_OC_), short-circuit current (*I*_SC_), and output power density is checked under similar experimental conditions with actual water wave conditions. The durability of the energy harvester is also analyzed with a long-term operating test. The applicability of the device is demonstrated by charging capacitors and illuminating light-emitting diodes (LEDs). Finally, an experiment in an artificial water wave condition is conducted to confirm the potential for harvesting water wave energy with the DR-TENG. With the simple structural modification, this fabricated DR-TENG can improve the devices’ space efficiency while enhancing electrical output from the same covered area on the ocean by the TENGs.

## 2. Materials and Methods

### 2.1. Fabrication of the DR-TENG

The frame of the DR-TENG was fabricated by a 3D printer (Cubicon 3DP-310F, Seongnam, Korea) using acrylonitrile butadiene styrene (ABS, ABS-A100) filament. The fabricated device with a volume of 108.2 cm^3^ was encapsulated with 2 mm-thick acrylic plate and attached with silicone hot-melt glue. For the electrode layers, rectangular (40 mm × (depth − 2 mm)) Al tape was attached to each side. The small and large rollers were 3D printed with the 4 diameter pairs, and gaps for the small and large roller tracks were 6.75 mm and 11 mm, with a medium curvature. The entire schematic of the optimized device is illustrated in Figure S8.

### 2.2. Surface Characterization of the DR-TENG Device

Nikon Eclipse LV100 (Nikon, Tokyo, Japan) was used for checking the microscopic surface structure of the untreated and acetone-treated ABS samples. The static contact angle was measured with SmartDrop (Femtobiomed, Seongnam, Korea).

### 2.3. FEM Analysis

COMSOL Multiphysics (COMSOL Inc., Stockholm, Sweden) was used to analyze the electric potential distribution with different numbers and types of rollers while changing the location of the rollers. The module of electromechanics was selected for implementing the TENG in finite element method (FEM). The surface charge density values on the surface of the rollers were selected to remain constant with the different roller locations. The surface charge density at the contact surfaces of the polyimide (PI) and contact electrodes were −1 × 10^−7^ C m^−2^ and 1 × 10^−7^ C m^−2^, respectively. The non-contact electrode was selected as the ground. Free triangular mesh with normal element size was adopted to divide the surface.

### 2.4. Measurement of the Electrical Output from the DR-TENG

A custom-made rotating machine with a frequency controllable alternating current (AC) motor was used to apply angular displacement to the DR-TENG, as shown in Video S1. The electrical outputs of the open-circuit voltage and short-circuit current were measured by a system electrometer (Keithley Model 6514, Keithley Instruments, LLC., Cleveland, OH, USA) through a DAQ system (NI PCI-6220, National Instruments Corporation, Austin, TX, USA). The measurement setup for measuring the basic electrical outputs is shown in Figure S1.

### 2.5. Setting Up a Water Wave Test

The DR-TENG device entirely covered with the silicone hot-melt glue layer was placed in the center point of a transparent glass bath thus that both crest and trough states could be applied to the fabricated device. The size of the glass bath was 1190 mm (width) × 430 mm (depth) × 430 mm (height), and the bath was filled with tap water to half of its maximum height. A frequency and intensity controllable propeller was used to generate the water wave with a pulse-type input in the bath. Two black wires were used to fix the location of the DR-TENG in the left-to-right direction of the water flow from the wave generator in Video S2. The waveform was matched to one cycle of the entire bath, and the measured frequency was in the range from 1 to 1.25 Hz. For checking more actual conditions in water waves, an anchor or the array structure was used to fix the location of the DR-TENG device instead of the fixed-line at the container. The water wave condition and the corresponding location of the 2 rollers can be seen in Figure S2.

## 3. Results

### 3.1. Structure and Operating Mechanism of the DR-TENG

**Figure 1:**
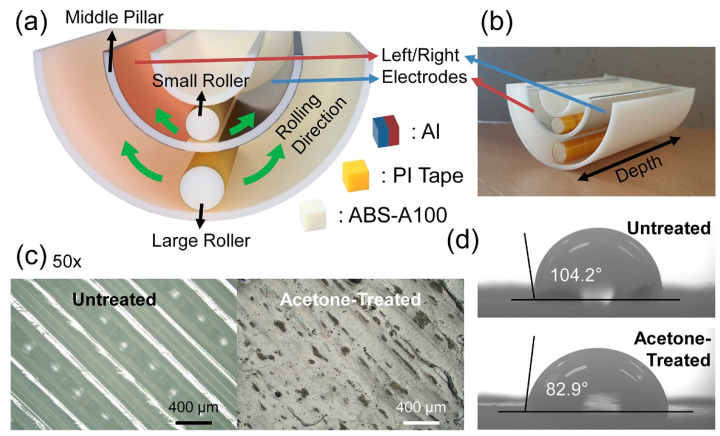
Structure and surface characteristics of the double roller-based triboelectric nanogenerator (DR-TENG) device. (**a**) Structural illustration of the DR-TENG. (**b**) Digital camera image of the entire device. (**c**) Optical microscope images of the untreated acrylonitrile butadiene styrene (ABS) surface and acetone-treated ABS surface. (**d**) Contact angle measurement results of the untreated and acetone-treated ABS cases.

The frame and rollers of the DR-TENG were fabricated with ABS filament using a 3D printer. Al tapes were attached to the left and right parts around the middle pillar between the inner pillar and outer pillar, as shown in Figure 1a. Two contacts were simultaneously established at an electrode/small roller and the same electrode/large roller. A PI tape was attached to the outside of the rollers for two reasons. First, the PI was employed to increase the triboelectric charges on the contact surface, according to the triboelectric series [42]. In the triboelectric series, Al is easy to be positively charged, whereas PI tends to be more negatively charged. In comparison to the distance between ABS and Al in the series, PI and Al show a farther distance due to the less negatively charging characteristic of ABS than that of PI. With adapting the contact pair with PI and Al, the DR-TENG can generate increased electrical outputs than in ABS and Al-case. In addition, the 3D printed surface, which has a bumpy state due to the 0.2 mm-resolution of the printing equipment, can be covered with this smooth PI tape. The smoother surface of the PI layer than the printed ABS surface allowed the rollers to more easily oscillate between left and right with decreased frictional loss. A digital camera image of the DR-TENG is displayed in Figure 1b indicating the left/right electrodes and small/large rollers.

For establishing the stable movement of the rollers in actual water wave conditions, the surface of the 3D printed device needs to be more soaked in water and be flattened. In Figure 1c, optical microscope images are displayed with the untreated ABS surface and acetone-treated ABS surface. The acetone-treatment process was conducted by dipping the 3D printed ABS surface in the acetone for 30 s and drying the surface with the N_2_ gas. The untreated ABS surface at the left side of Figure 1c presents the air-gap of 140 µm between the two filament lines. However, the acetone-treated ABS surface at the right side of Figure 1c shows no obvious air gap. This result can be quantitatively confirmed by measuring the static contact angle of the two samples. The microscopic images of the 5 µL-droplet of the deionized (DI) water on each surface are shown in Figure 1d. With the flattened surface after the acetone treatment process, the acetone-treated ABS showed a smaller contact angle of 82.9° than that in the untreated ABS of 104.2°. More hydrophilic characteristic of the acetone-treated ABS can also be proved with this contact angle measurement. Furthermore, as shown in Figure S3, more hydrophilic characteristics of the acetone-treated ABS surface can be proven with the water floating test. Two iceberg-like cubes with a height of 14.5 mm were 3D printed, and only one sample was treated with acetone. The depth of the submerged structure on water is deeper in the acetone-treated case with 6 mm than that of 4.5 mm from the untreated case. Through the acetone-treatment process, the 3D printed ABS surface with the hydrophilic characteristic was successfully attained, and the more stable water wave energy harvester can be fabricated.

**Figure 2:**
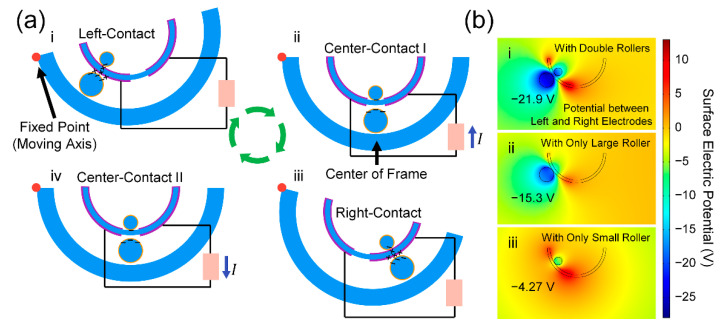
Working mechanism of the DR-TENG. (**a**) Operating principle of the DR-TENG with the double side-covered electrodes and double rollers. (**b**) Finite element method (FEM) simulation result indicating the surface electric potential with double rollers, only one large roller, and only one small roller.

The DR-TENG operates based on the simple pendulum motion of two rollers. The top left side of the device is set as a fixed point of a moving axis. Figure 2(ai) shows the initial state of the DR-TENG with the highest point of the top right side of the device. The contact electrification is generated at the contact surfaces, with the negatively charged two PI layers and the positively charged left Al layer. When the frame of the device rotates in a clockwise direction, both rollers move to the center of the frame, as shown in Figure 2(aii). In this state, the current flows from the left to the right electrode in the same moving direction of positive charges. The rollers move to the right side of the frame when the top right side of the device reaches the lowest point in Figure 2(aiii). The right electrode is positively charged in this state. In Figure 2(aiv), after the frame of the device rotates in a counter-clockwise direction, the rollers move back to the center of the frame with the right-to-left direction of the current flowing. These four states are repeated by the reciprocating rotation of the frame, and a cycle of the alternating current is generated to the external load through these four states.

For a single roller-case shown in Figure S4, the operating principle is simpler than the double roller-case. In Figure S4a, the roller and the left electrode are in contact. When an external force is applied, and the frame rotates in a clockwise direction, these rollers and left electrodes are separated with the changed location of the roller at the center of the frame structure in Figure S4b. Electrical current flows from the left electrode to the right electrode. The roller is in contact with the right electrode after stopping the movement in Figure S4c. When a counter-clockwise rotation of the frame occurs, the roller moves back toward the left electrode with an opposite flowing current from the right electrode to the left electrode in Figure S4d. In this single roller mechanism, the number of charges accumulated on or flowing through the electrodes would be less than that of the double roller mechanism due to the diminished contact surface area. This tendency can be observed in the following simulation results.

FEM simulations were conducted to analyze the intensity of the surface electrical potential that could be achieved from contact electrification, varying the number of rollers. The electrical potential difference between the two electrodes represented −21.9 V with the double rollers, −15.3 V with only the large roller, and −4.27 V with only the small roller, as shown in Figure 2(bi–iii). Increasing the number of rollers and using the larger size of the roller resulted in a higher difference in electric potential because the contact surface area between the roller and electrode was enlarged. In the same manner, described above for the operating mechanism, the surface electric potential distributions with changing the locations of the two rollers at the center point and the right side of the frame are represented with values of 0 V and +21.9 V, respectively, in Figure S5.

### 3.2. Structure and Operating Mechanism of the DR-TENG

**Figure 3:**
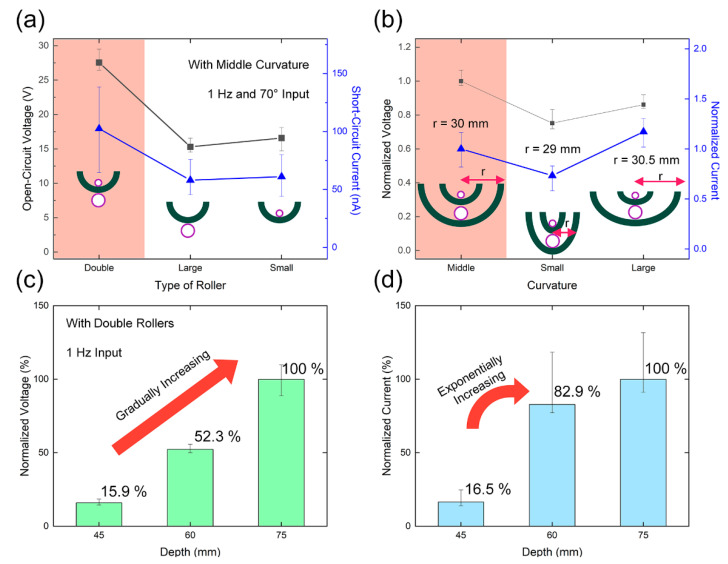
Structural optimization of the fabricated DR-TENG from electrical output characteristics. (**a**) Open-circuit voltages (*V*_OC_s) and short-circuit currents (*I*_SC_s) from the three cases, with double rollers, only one large roller, and only one small roller. (**b**) *V*_OC_s and *I*_SC_s with the three different curvatures. (**c**,**d**) Normalized electrical outputs with three different depths.

The structural parameters of the DR-TENG device were changed to determine the optimized structure. In Figure 3a, the highest values of both the open-circuit voltage (*V*_OC_) of 27.6 V and short-circuit current (*I*_SC_) of 102.5 nA were obtained for the double roller structure without any external circuit. While the small and large rollers moved in physically individual states, the accumulated charges at the contact electrode did not cancel each other because the rollers had the same period of movement with identical contact-separation timing. Unlike the FEM result in Figure 2b, the higher electrical outputs of the only small roller-case were caused by higher contact force with the electrode layer from the direct contact mode than in the non-contact mode of the large roller. Due to the location of the small roller, the contact electrode was compressed by the gravitational force of the small roller.

When the gap distance was fixed to 6.75 mm for the small roller and 11 mm for the large roller, the diameters of the rollers were changed to attain the optimized electrical outputs with the 3 Hz input. The sets with the 4.5/8.5 (case 1), 5/9 (case 2), 5.5/9.5 (case 3), 6/10 (case 4), and 5/9.5 (case 5) were used. The former number and latter number are indicating the diameters (mm) of the small and large rollers, respectively. Both normalized electrical outputs of the *V*_OC_ and *I*_SC_ displayed a similar tendency with varying diameters in Figure S6a,b, respectively. Case 3 with 5.5 mm/9.5 mm showed the optimum electrical outputs. With the small size of the rollers, the distance between the electrode layers and the large roller can decrease the electrostatic induction on the electrode layers compared to case 1 to case 4. Even though the contact area between the small roller remains a point contact, the contact area was slightly enlarged with increasing the diameter of the small roller. This result can be verified by comparing case 3 and case 5. Case 4 showed the lowest output due to the interruption of the roller movement with the increased diameters. The space for the rollers needs to be secured over 1.25 mm of the roller diameter.

The effect of the electrode structures was also studied by measuring electrical outputs with three electrode conditions. Only inner electrodes (case A), only outer electrodes (case B), and double side-covered and connected electrodes (case A + B) were used to confirm the output characteristics with the normalized *V*_OC_ and *I*_SC_ in Figure S7a. The values for single electrode area of the case A and B were 75 mm × 20 mm and 75 mm × 25 mm, respectively. The larger electrode area of case B can generate higher outputs by inducing more charges at the electrode surface than in case A in Figure S7b. Moreover, the case A + B showed the highest electrical outputs than in the case A and B due to a synergistic effect through matching the contact-separation timing of the electrode and rollers. It is notable that the electrical outputs can be enhanced from 8 to 62% by simply adding electrode layers without enlarging the device area. Additional electrical output can be generated by utilizing the surface under the large roller and above the small roller as electrodes.

Next, as structural optimization, the curvature of the frame was changed, and the resulting electrical output characteristics are shown in Figure 3b. A small curvature of *κ* = 0.0345 mm^−1^, a middle curvature of *κ* = 0.0333 mm^−1^, and a large curvature of *κ* = 0.0328 mm^−1^ were adopted as experimental samples with a fixed frequency of 1 Hz. The middle curvature case showed optimum electrical outputs compared with the two other cases. The small curvature exhibited the smallest output because of the shorter lateral space of the frame where the rollers accelerate in their pendulum motion. The large curvature displayed the largest *I*_SC_ because of the longer acceleratable space for the rollers at the frame. However, the decreased *V*_OC_ than in the middle curvature showed that the duration of the flight with the small roller was increased without causing contact with the electrodes.

The depth of both the frame and the rollers was varied from 45 mm to 75 mm at intervals of 15 mm, and both the normalized output voltage and current were increased with the enlarged contact area between the rollers and electrodes in Figure 3c,d. The *I*_SC_ showed a little saturation tendency distinguished from the *V*_OC_ case due to the shorter displacement during the same moving time with a heavier weight of the longer roller over 60 mm-depth. After considering all the factors, the double rollers, double side-covered electrodes, middle curvature, and 75 mm-depth were selected for the optimized structure and used in the following experiments. The final optimized structure and its structural parameters are presented in Figure S8.

### 3.3. Electrical Output Characteristics of the DR-TENG

**Figure 4:**
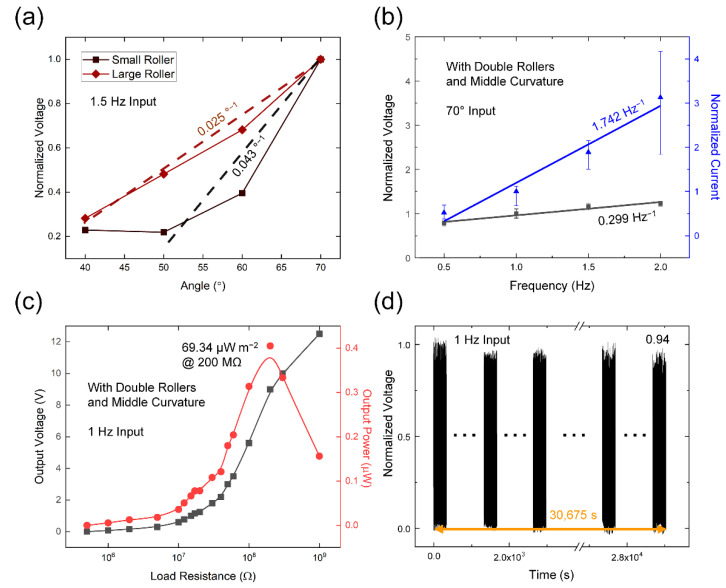
Electrical output characteristics of the DR-TENG. Normalized voltages with (**a**) different angular displacement and (**b**) different frequency. (**c**) Output power with both double rollers and middle curvature case. (**d**) Durability test with 8.5 h operation.

Various experimental conditions were analyzed in an effort to achieve the electrical output characteristics of the DR-TENG. The dependency on displacement angle of the top right side of the device was firstly checked with varying angles from 40° to 70° at 10° intervals. As a result, the output voltages of both the small and large roller cases were increased with the higher displacement angle because the longer displacement of the rollers was generated, and a more distinctive contact separation between the rollers and electrodes was generated, shown in Figure 4a. The small roller showed a higher gradient of 0.0043°^−1^ than the large roller, with 0.0025°^−1^. Since, when the same input force is applied to the small roller and the large roller, the acceleration of the small roller with a lower weight will show a higher value than that of the large roller. With the double rollers, the output current and voltage showed a similar rising tendency with increasing the displacement angle in Figure S9. Under 30°, the displacement of both the rollers was relatively small, leading to undetectable electrical output values.

The frequency responses of the DR-TENG outputs were checked with changing input frequencies from 0.5 to 2.0 Hz at intervals of 0.5 Hz. In Figure 4b, the gray line and blue line indicate the linear and nonlinear fitting curves of the output voltage and current, respectively. Both the output voltage and current which was normalized to each 1 Hz output, gradually escalated with heightening the input frequency and were shown the highest values of 1.224 and 3.128 at the 2 Hz outputs, respectively. Comparing the gradient of the linear fitting results, the *I*_SC_ with 1.742 Hz^−1^ was increased faster than that of *V*_OC_ with the value of 0.299 Hz^−1^ by growing the frequency. This result was caused by the proportionality of the *I*_SC_ and velocity of the rollers, given the linear relationship between *I*_SC_ and the velocity of charge transfer [43]. The *V*_OC_ was slightly affected by the varying frequency due to the increased input force at the higher frequency from the direct current (DC) motor. By comparing the three different curvatures, the middle curvature case showed the highest gradient, indicating the highest delivered force to the rollers and the effective contact between the rollers and electrode in Figure S10. In the low-frequency range under 1 Hz, both the electrical outputs were highest in the large curvature case. The two rollers can easily move between the two electrodes with lower input force at the large curvature case, but the electrical outputs were saturated faster than other samples.

The output power density of the DR-TENG was 69.34 µW m^−2^ at 200 MΩ with an electrode area of 58 cm^2^, as shown in Figure 4c. This electrical power value was calculated by dividing the squared output voltage by the load resistance value. The output voltage was gradually increased with a growing load resistance value approaching the open-circuit condition. Using the maximum power transfer theorem, the input resistance of the DR-TENG device was determined to be identical to the load resistance, with 200 MΩ showing the highest output power. In a durability test shown in Figure 4d, the device stably generated output voltage during 30,675 s with only a 0.06 decrease in the normalized voltage.

### 3.4. Applications of the DR-TENG

**Figure 5:**
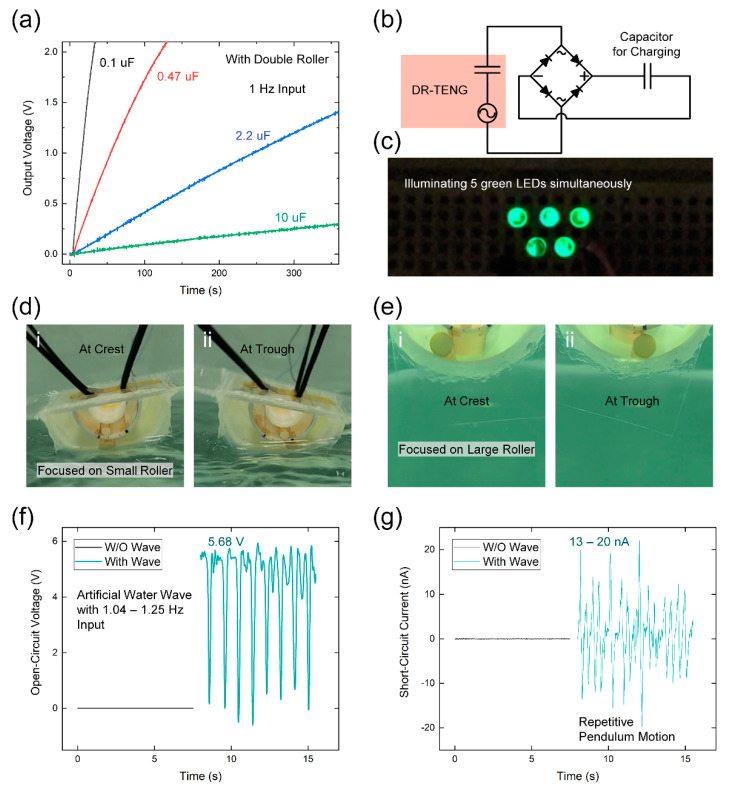
Applications of the proposed DR-TENG. (**a**) Charging characteristic of capacitors with four different capacitances. (**b**) Circuit diagram for charging the capacitor. (**c**) Simultaneously illuminating five green LEDs. (**d**,**e**) Digital camera images of the DR-TENG during the test with the artificial water wave with two different focuses. (**f**) *V*_OC_ and (**g**) *I*_SC_ from the water wave test.

To evaluate its use in practical applications, the optimized DR-TENG was operated to charge capacitors, illuminate LEDs, and test in a water bath. As shown in Figure 5a, the DR-TENG was used to charge 0.1, 0.47, 2.2, and 10 µF capacitors. Figure 5b illustrates the circuit diagram used for charging the capacitors with the DR-TENG through a full-wave bridge rectifier. 33.2 s and 130.0 s were required to charge the 0.1 and 0.47 µF capacitors, respectively, at 2.1 V with 1 Hz and 70°-input. After charging the capacitors with DR-TENG during 360 s, the 2.2 and 10 µF capacitors can be charged to 1.4 V and 0.3 V, respectively. The charging rate was higher when using the capacitors with lower capacitance due to their lower capacity. Five serially connected commercial green LEDs were simultaneously illuminated without charging or any external circuit when the DR-TENG was operating at 1 Hz and 70°-input in Figure 5c and Video S1. This ability to light five LEDs can indirectly represent the output power of the DR-TENG.

The capacity for blue energy harvesting with the DR-TENG is shown in Figure 5d–g. The entire device floated in the water bath, and black wires were used to fix its location for capturing the movement of the rollers in the water wave condition. Figure 5d,e displays the fully encapsulated DR-TENG structure and the movement of the rollers in the water bath, separately focusing on the small roller and large roller. The frequency of the water wave input was randomly applied with a range between 1.0 and 1.25 Hz. In Figure 5d,e, the first (i) and second (ii) figures represent the DR-TENG states at the crest leaning to the left side and trough leaning to the right side, respectively. The continuous motion of the rollers in the floating DR-TENG can be checked in Video S2. The repetitive leaning movement of the DR-TENG can generate a *V*_OC_ of 5.68 V and *I*_SC_ with a range of 13 to 20 nA, as presented in Figure 5f,g, respectively. To distinguish this electrical output from the background noise by the outer water, the conditions before and after injecting the water wave were compared, and there were no detected *V*_OC_ and *I*_SC_ signals before applying the wave. Therefore, even though the electrical output levels were decreased in comparison with the outputs using the DC motor, the real signal was detected and can be increased with suitable fixing methods. By comparing the electrical outputs from the non-hydrophilic and hydrophilic ABS cases in the water wave condition, the higher output voltage was shown in the hydrophilic ABS case in Figure S11. With the hydrophilic ABS, the movement of the water wave can be regularly transferred by minimizing the floating height over the surface level of the water. Moreover, the output stability can be improved for the aforementioned reason.

For harvesting energy in the actual water wave condition, instead of the fixed wire, an anchor was hung under the DR-TENG device. One LED can be operated by connecting an anchor, and this actual condition is shown in Video S3. However, even if there is no connection between the DR-TENG device and water container, this case with using the anchor was difficult to be seen as completely freestanding. Therefore, another method for constituting the array structure was introduced to fix the location of the DR-TENG and enhance the output stability. The 2 × 2 array structure was adopted to show the ability for the integration of the DR-TENG. The four DR-TENG units were fixed under the 300 µm-thick poly (ethylene terephthalate) (PET) film. This DR-TENG array can operate in a water wave condition without any additional structure such as wire or anchor shown in Video S4. The durability of the DR-TENG with the water wave condition was also verified by using the array structure. With injecting the wave energy, 95.7% of the initial voltage was confirmed after the continuous operation of 7 h, representing the stable output as shown in Figure S12. The potential utility of the DR-TENG for the blue energy harvesting in a large area was provided with the implementation of this array structure.

## 4. Discussion

In this study, a freestanding mode DR-TENG with the acetone-treated ABS surface and double side-covered electrodes between double rollers was studied to investigate its potential for harvesting wave energy. The operating principle of the DR-TENG was elucidated, showing the triboelectric charges and flow of charges. A finite element method was also conducted to visualize the surface electric potential distribution, using different sizes and numbers of rollers. To attain the highest electrical outputs with the 3D printed DR-TENG, the structural parameters of curvature and number of rollers were fixed with a curvature of 0.0333 mm^−1^ and double rollers, respectively. When high moving angle and high frequency were used as experimental conditions, it resulted in increased electrical outputs. The double side-covered electrodes represented enhanced electrical outputs than the single-electrode cases. *V*_OC_ of 27.6 V, *I*_SC_ of 102.5 nA, and power density of 69.34 µW m^−2^ at 200 MΩ were harvested with this optimized DR-TENG structure. The durability of the DR-TENG was also examined and only a 6% decrease was exhibited after 8.5 h operation. 33.2 s was required to charge a 0.1 µF capacitor with 2.1 V, and five LEDs were simultaneously illuminated with the DR-TENG. The potential for using the DR-TENG as a blue energy harvester was confirmed by testing with an injected 1 Hz to 1.25 Hz water wave in the conditions with the wires, anchor, and array structure. Although a structural modification with the output optimizing process is required, the output can be enhanced with the introduction of the double side-covered electrodes and double rollers. This DR-TENG can be used as a water energy harvester with enhanced contact area and space conservative method for ecologically friendly technology.

## Supplementary Materials

The following are available online at https://www.mdpi.com/article/10.3390/mi12091089/s1, Figure S1: measurement condition for the basic electrical outputs, Figure S2: water wave conditions and movement of the two rollers, Figure S3: water floating test for checking hydrophilicity of acetone-treated ABS, Figure S4: operating principle of single roller-TENG, Figure S5: FEM simulation results at different location of the rollers, Figure S6: effect of the roller diameters in the DR-TENG, Figure S7: structure and electrical outputs by varying the electrode structures, Figure S8: optimized structure and its parameters, Figure S9: angular dependency of the DR-TENG, Figure S10: frequency response of the DR-TENG with three curvatures, Figure S11: output comparison with and without the hydrophilic treatment-cases, Figure S12: result of durability test in the water wave condition, Video S1: video of illuminating LEDs with DR-TENG, Video S2: video of water wave harvesting test in a water bath, Video S3: video of water wave harvesting test in the actual water wave condition with an anchor and operating a LED, and Video S4: video of water wave harvesting test with array structure in the water wave condition.

## References

[1] Jin Z., Charlock T.P., Smith W.L., Rutledge K. (2004). A Parameterization of Ocean Surface Albedo. Geophys. Res. Lett..

[2] Höök M., Tang X. (2013). Depletion of Fossil Fuels and Anthropogenic Climate Change-A Review. Energy Policy.

[3] Chiari L., Zecca A. (2011). Constraints of Fossil Fuels Depletion on Global Warming Projections. Energy Policy.

[4] Guler U., Sendi M.S.E., Ghovanloo M. A Dual-Mode Passive Rectifier for Wide-Range Input Power Flow. Proceedings of the 2017 IEEE 60th International Midwest Symposium on Circuits and Systems (MWSCAS).

[5] Batten W.M.J., Bahaj A.S., Molland A.F., Chaplin J.R. (2007). Experimentally Validated Numerical Method for the Hydrodynamic Design of Horizontal Axis Tidal Turbines. Ocean. Eng..

[6] Myers L.E., Bahaj A.S. (2010). Experimental Analysis of the Flow Field around Horizontal Axis Tidal Turbines by Use of Scale Mesh Disk Rotor Simulators. Ocean. Eng..

[7] Viviano A., Naty S., Foti E., Bruce T., Allsop W., Vicinanza D. (2016). Large-Scale Experiments on the Behaviour of a Generalised Oscillating Water Column under Random Waves. Renew. Energy.

[8] Guarnieri M. (2018). The Development of Ac Rotary Machines. IEEE Ind. Electron. Mag..

[9] Träsch M., Déporte A., Delacroix S., Drevet J.B., Gaurier B., Germain G. (2018). Power Estimates of an Undulating Membrane Tidal Energy Converter. Ocean. Eng..

[10] Fan F.R., Tian Z.Q., Wang Z.L. (2012). Flexible Triboelectric Generator. Nano Energy.

[11] Wang Z.L., Chen J., Lin L. (2015). Progress in Triboelectric Nanogenerators as a New Energy Technology and Self-Powered Sensors. Energy Environ. Sci..

[12] Wu C., Wang A.C., Ding W., Guo H., Wang Z.L. (2019). Triboelectric Nanogenerator: A Foundation of the Energy for the New Era. Adv. Energy Mater..

[13] Kim D., Oh Y., Hwang B.W., Jeon S.B., Park S.J., Choi Y.K. (2016). Triboelectric Nanogenerator Based on the Internal Motion of Powder with a Package Structure Design. ACS Nano.

[14] Guo T., Liu G., Pang Y., Wu B., Xi F., Zhao J., Bu T., Fu X., Li X., Zhang C. (2018). Compressible Hexagonal-Structured Triboelectric Nanogenerators for Harvesting Tire Rotation Energy. Extrem. Mech. Lett..

[15] Kim D., Tcho I.W., Choi Y.K. (2018). Triboelectric Nanogenerator Based on Rolling Motion of Beads for Harvesting Wind Energy as Active Wind Speed Sensor. Nano Energy.

[16] Kim I., Chae Y., Jo S., Kim D. (2020). Levitating Oscillator-Based Triboelectric Nanogenerator for Harvesting from Rotational Motion and Sensing Seismic Oscillation. Nano Energy.

[17] Gao S., Zhu Y., Chen Y., Tian M., Yang Y., Jiang T., Wang Z.L. (2019). Self-Power Electroreduction of N2 into NH3 by 3D Printed Triboelectric Nanogenerators. Mater. Today.

[18] Tian M., Zhang D., Wang M., Zhu Y., Chen C., Chen Y., Jiang T., Gao S. (2020). Engineering Flexible 3D Printed Triboelectric Nanogenerator to Self-Power Electro-Fenton Degradation of Pollutants. Nano Energy.

[19] Peng X., Dong K., Ye C., Jiang Y., Zhai S., Cheng R., Liu D., Gao X., Wang J., Wang Z.L. (2020). A Breathable, Biodegradable, Antibacterial, and Self-Powered Electronic Skin Based on All-Nanofiber Triboelectric Nanogenerators. Sci. Adv..

[20] Dong K., Peng X., Wang Z.L. (2020). Fiber/Fabric-Based Piezoelectric and Triboelectric Nanogenerators for Flexible/Stretchable and Wearable Electronics and Artificial Intelligence. Adv. Mater..

[21] Dong K., Peng X., An J., Wang A.C., Luo J., Sun B., Wang J., Wang Z.L. (2020). Shape Adaptable and Highly Resilient 3D Braided Triboelectric Nanogenerators as E-Textiles for Power and Sensing. Nat. Commun..

[22] Peng X., Dong K., Wu Z., Wang J., Wang Z.L. (2021). A Review on Emerging Biodegradable Polymers for Environmentally Benign Transient Electronic Skins. J. Mater. Sci..

[23] Rodrigues C., Kumar M., Proenca M.P., Gutierrez J., Melo R., Pereira A., Ventura J. (2020). Triboelectric Energy Harvesting in Harsh Conditions: Temperature and Pressure Effects in Methane and Crude Oil Environments. Nano Energy.

[24] Kim I., Roh H., Yu J., Jayababu N., Kim D. (2020). Boron Nitride Nanotube-Based Contact Electrification-Assisted Piezoelectric Nanogenerator as a Kinematic Sensor for Detecting the Flexion–Extension Motion of a Robot Finger. ACS Energy Lett..

[25] Seol M.L., Han J.W., Moon D.I., Meyyappan M. (2017). Triboelectric Nanogenerator for Mars Environment. Nano Energy.

[26] Liu L., Shi Q., Ho J.S., Lee C. (2019). Study of Thin Film Blue Energy Harvester Based on Triboelectric Nanogenerator and Seashore IoT Applications. Nano Energy.

[27] Chen X., Gao L., Chen J., Lu S., Zhou H., Wang T., Wang A., Zhang Z., Guo S., Mu X. (2020). A Chaotic Pendulum Triboelectric-Electromagnetic Hybridized Nanogenerator for Wave Energy Scavenging and Self-Powered Wireless Sensing System. Nano Energy.

[28] Chen J., Yang J., Li Z., Fan X., Zi Y., Jing Q., Guo H., Wen Z., Pradel K.C., Niu S. (2015). Networks of Triboelectric Nanogenerators for Harvesting Water Wave Energy: A Potential Approach toward Blue Energy. ACS Nano.

[29] Liang X., Jiang T., Feng Y., Lu P., An J., Wang Z.L. (2020). Triboelectric Nanogenerator Network Integrated with Charge Excitation Circuit for Effective Water Wave Energy Harvesting. Adv. Energy Mater..

[30] Liang X., Jiang T., Liu G., Feng Y., Zhang C., Wang Z.L. (2020). Spherical Triboelectric Nanogenerator Integrated with Power Management Module for Harvesting Multidirectional Water Wave Energy. Energy Environ. Sci..

[31] Liu G., Guo H., Xu S., Hu C., Wang Z.L. (2019). Oblate Spheroidal Triboelectric Nanogenerator for All-Weather Blue Energy Harvesting. Adv. Energy Mater..

[32] Wang X., Niu S., Yin Y., Yi F., You Z., Wang Z.L. (2015). Triboelectric Nanogenerator Based on Fully Enclosed Rolling Spherical Structure for Harvesting Low-Frequency Water Wave Energy. Adv. Energy Mater..

[33] Gao L., Lu S., Xie W., Chen X., Wu L., Wang T., Wang A., Yue C., Tong D., Lei W. (2020). A Self-Powered and Self-Functional Tracking System Based on Triboelectric-Electromagnetic Hybridized Blue Energy Harvesting Module. Nano Energy.

[34] Xie Z., Dong J., Li Y., Gu L., Song B., Cheng T., Wang Z.L. (2020). Triboelectric Rotational Speed Sensor Integrated into a Bearing: A Solid Step to Industrial Application. Extrem. Mech. Lett..

[35] Yang W., Gao Q., Xia X., Zhang X., Lu X., Yang S., Cheng T., Wang Z.L. (2020). Travel Switch Integrated Mechanical Regulation Triboelectric Nanogenerator with Linear–Rotational Motion Transformation Mechanism. Extrem. Mech. Lett..

[36] Saadatnia Z., Asadi E., Askari H., Esmailzadeh E., Naguib H.E. (2018). A Heaving Point Absorber-Based Triboelectric-Electromagnetic Wave Energy Harvester: An Efficient Approach toward Blue Energy. Int. J. Energy Res..

[37] Xu M., Zhao T., Wang C., Zhang S.L., Li Z., Pan X., Wang Z.L. (2019). High Power Density Tower-like Triboelectric Nanogenerator for Harvesting Arbitrary Directional Water Wave Energy. ACS Nano.

[38] Hao C., He J., Zhai C., Jia W., Song L., Cho J., Chou X., Xue C. (2019). Two-Dimensional Triboelectric-Electromagnetic Hybrid Nanogenerator for Wave Energy Harvesting. Nano Energy.

[39] Wang X., Wen Z., Guo H., Wu C., He X., Lin L., Cao X., Wang Z.L. (2016). Fully Packaged Blue Energy Harvester by Hybridizing a Rolling Triboelectric Nanogenerator and an Electromagnetic Generator. ACS Nano.

[40] Wu Z., Guo H., Ding W., Wang Y.C., Zhang L., Wang Z.L. (2019). A Hybridized Triboelectric-Electromagnetic Water Wave Energy Harvester Based on a Magnetic Sphere. ACS Nano.

[41] Ahmed A., Saadatnia Z., Hassan I., Zi Y., Xi Y., He X., Zu J., Wang Z.L. (2017). Self-Powered Wireless Sensor Node Enabled by a Duck-Shaped Triboelectric Nanogenerator for Harvesting Water Wave Energy. Adv. Energy Mater..

[42] Gooding D.M., Kaufman G.K. (2011). Tribocharging and the Triboelectric Series. Encyclopedia of Inorganic and Bioinorganic Chemistry.

[43] Niu S., Liu Y., Chen X., Wang S., Zhou Y.S., Lin L., Xie Y., Wang Z.L. (2015). Theory of Freestanding Triboelectric-Layer-Based Nanogenerators. Nano Energy.

