# Supplementary material for: 3D Printed Double Roller-Based Triboelectric Nanogenerator for Blue Energy Harvesting
Source: Micromachines (Basel). 2021 Sep 10;12(9):1089. doi: 10.3390/mi12091089 (PMC8469981; doi:10.3390/mi12091089)
Supplement: Supplementary file 1 [file micromachines-12-01089-s001.zip › micromachines-1371530-supplementary.pdf]

Supporting Information

3D Printed Double Roller-Based Triboelectric Nanogenerator for Blue Energy Harvesting

Inkyum Kim[1,2] and Daewon Kim[2,3,*]

[1] Department of Electronics and Information Convergence Engineering, Kyung Hee University, 1732 Deogyeong-daero, Giheung-gu, Yongin 17104, Republic of Korea
[2] Institute for Wearable Convergence Electronics, Kyung Hee University, 1732 Deogyeong-daero, Giheung-gu, Yongin 17104, Republic of Korea
[3] Department of Electronic Engineering, Kyung Hee University, 1732 Deogyeong-daero, Giheung-gu, Yongin 17104, Republic of Korea
* Correspondence: daewon@khu.ac.kr (D. Kim)

Table of contents

1. Measurement condition for the basic electrical outputs
2. Water wave conditions and movement of the two rollers
3. Water floating test for checking hydrophilicity of acetone-treated ABS
4. Operating principle of single roller-TENG
5. FEM simulation results at a different location of the rollers
6. Effect of the roller diameters in the DR-TENG
7. Structure and electrical outputs by varying the electrode structures
8. Optimized structure and its parameters
9. Angular dependency of the DR-TENG
10. Frequency response of the DR-TENG with three curvatures
11. Output comparison with and without the hydrophilic treatment-cases
12. Result of durability test in the water wave condition
13. Video of illuminating LEDs with DR-TENG
14. Video of water wave harvesting test in a water bath
15. Video of water wave harvesting test in the actual water wave condition with an anchor and operating a LED
16. Video of water wave harvesting test with array structure in the water wave condition

1. Measurement condition for the basic electrical outputs

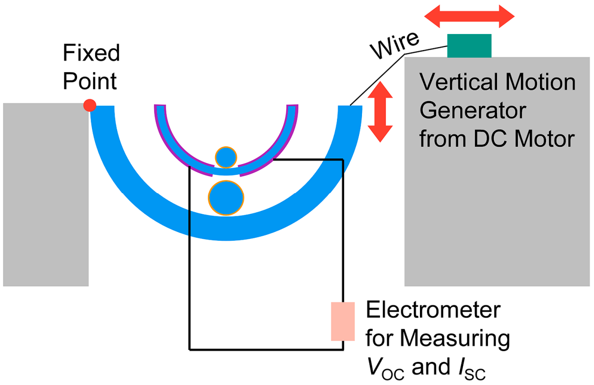

Figure S1. Schematic image for representing the measurement condition of the electrical output.

2. Water wave conditions and movement of the two rollers

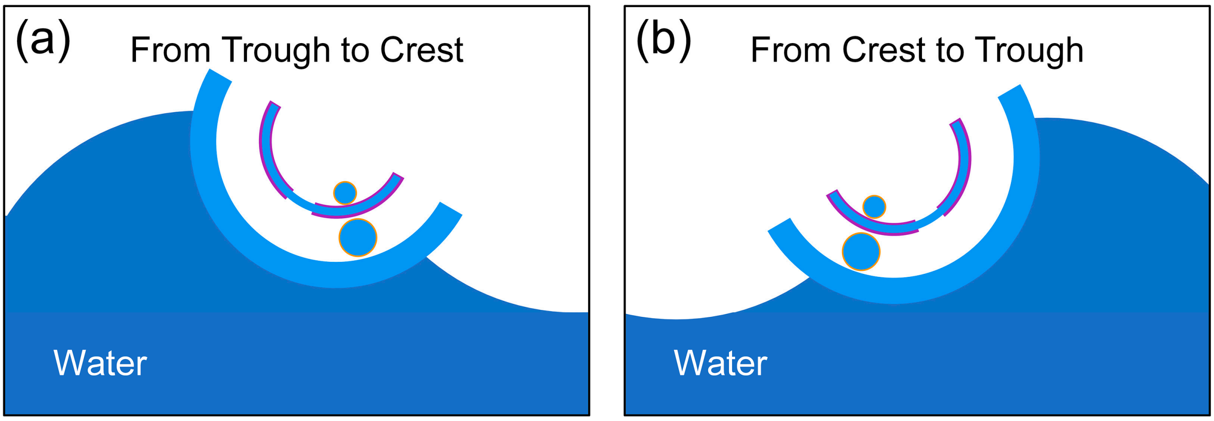

Figure S2. Illustrations for displaying the two water wave conditions of (a) from trough to crest and (b) from crest to trough, showing the location of the double rollers.

3. Water floating test for checking hydrophilicity of acetone-treated ABS

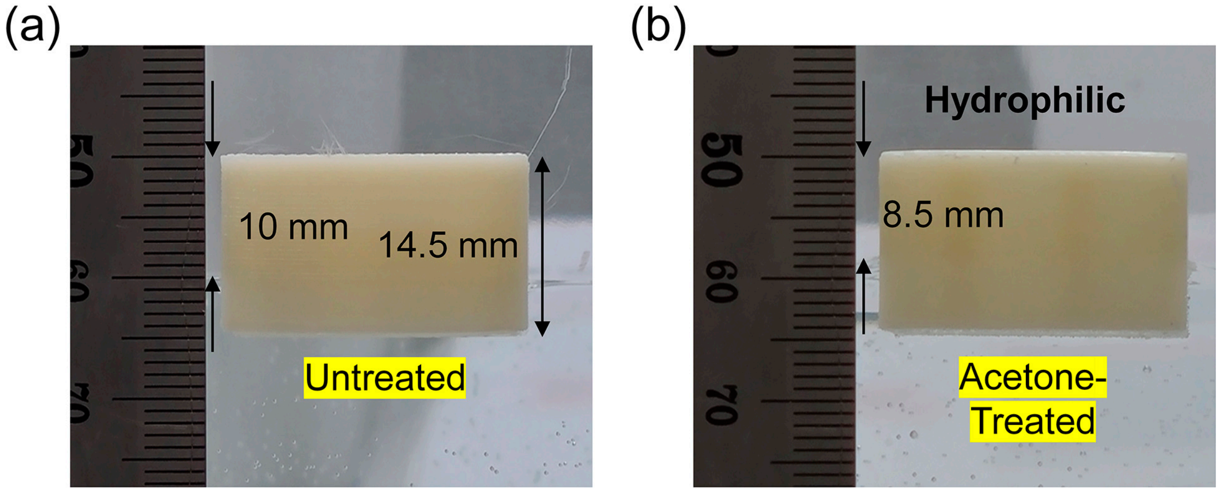

Figure S3. Digital camera images of (a) the untreated and (b) acetone-treated ABS cubes floating on water.

4. Operating principle of single roller-TENG

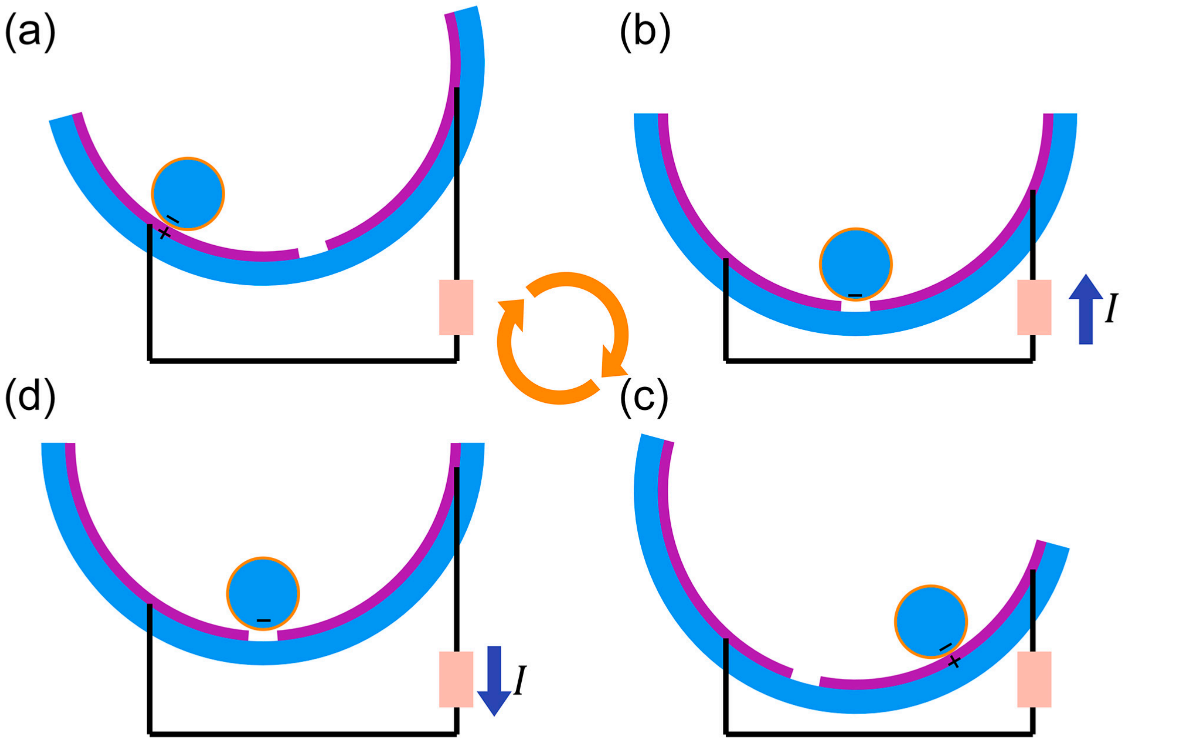

Figure S4. Operating principle of TENG using only one roller presenting a cycle from (a) left electrode-contact, (b) separation, (c) right electrode-contact, and (d) separation.

5. FEM simulation results at different location of the rollers

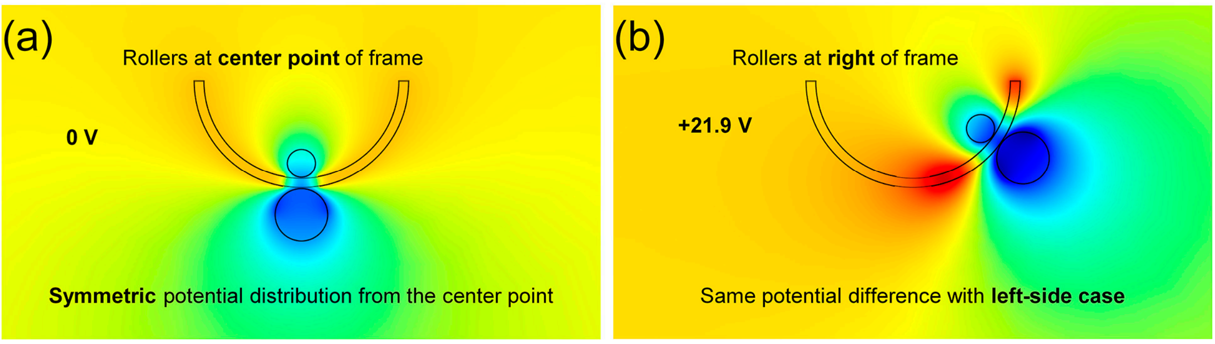

Figure S5. Surface electric potential distribution of the DR-TENG at (a) center position and (b) right of the frame.

6. Effect of the roller diameters in the DR-TENG

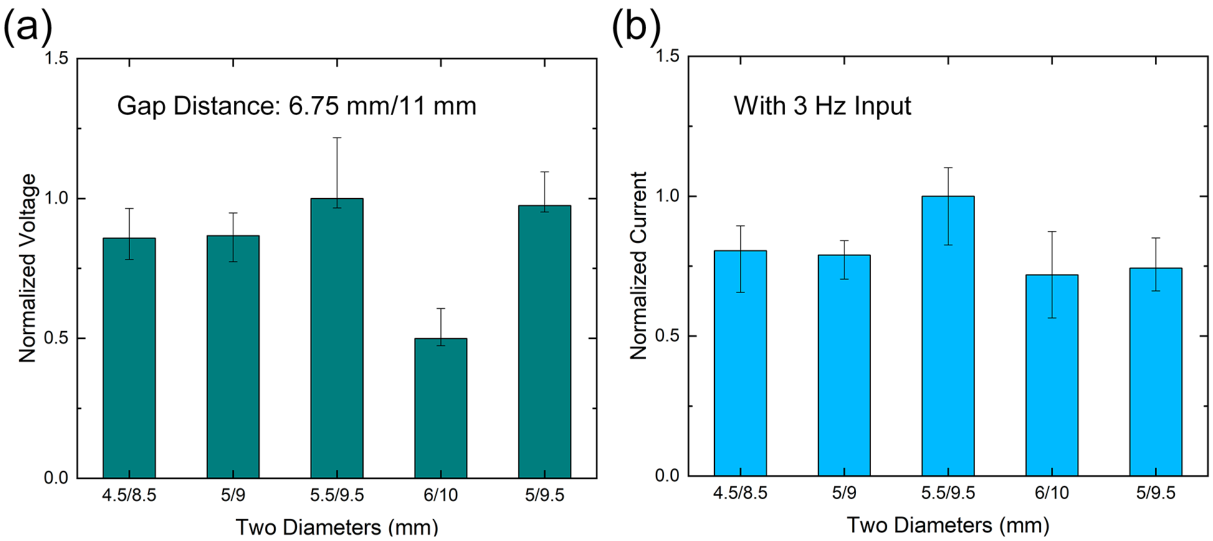

Figure S6. Effect on the (a) output voltage and (b) output current with changing the diameter of the two rollers.

7. Structure and electrical outputs by varying the electrode structures

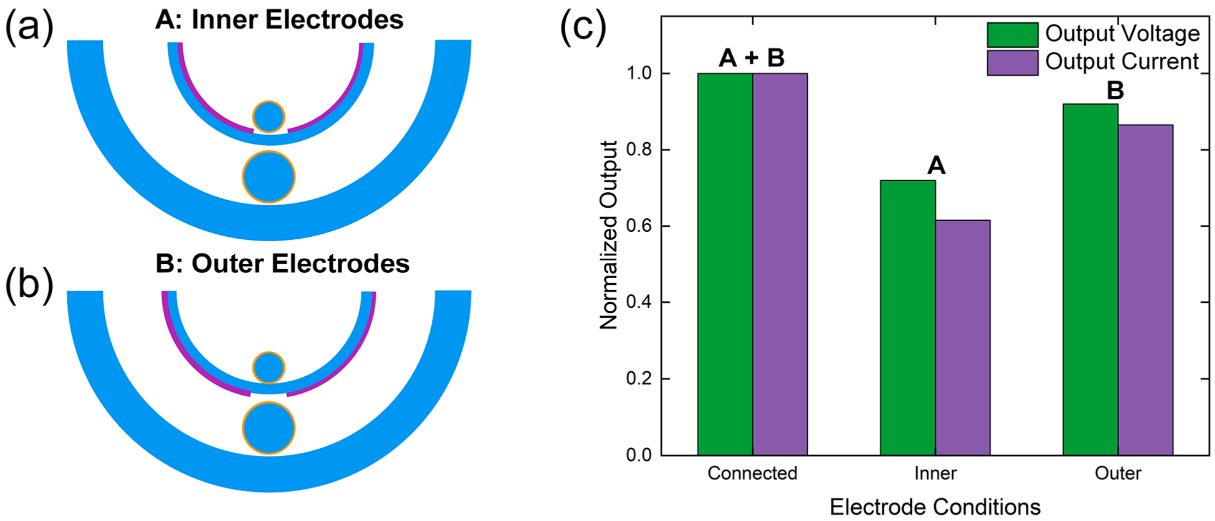

Figure S7. Electrode structures for (a) only inner electrodes and (b) only outer electrodes. (c) Normalized electrical outputs from three different electrode structures.

8. Optimized structure and its parameters

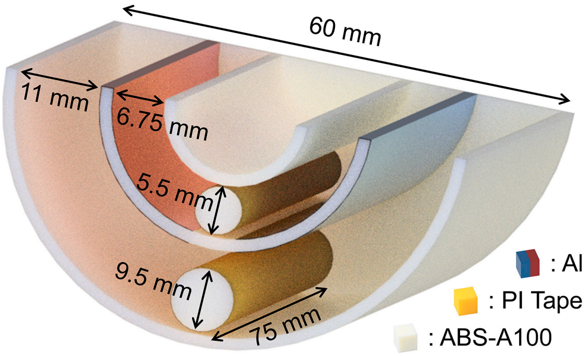

Figure S8. Schematic image for showing the structural parameters of the optimized DR-TENG.

9. Angular dependency of the DR-TENG

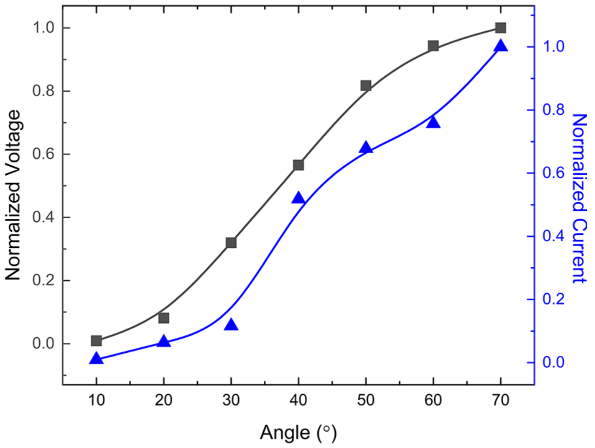

Figure S9. Normalized voltage and current with changing the displacement angle of the DR-TENG.

10. Frequency response of the DR-TENG with three curvatures

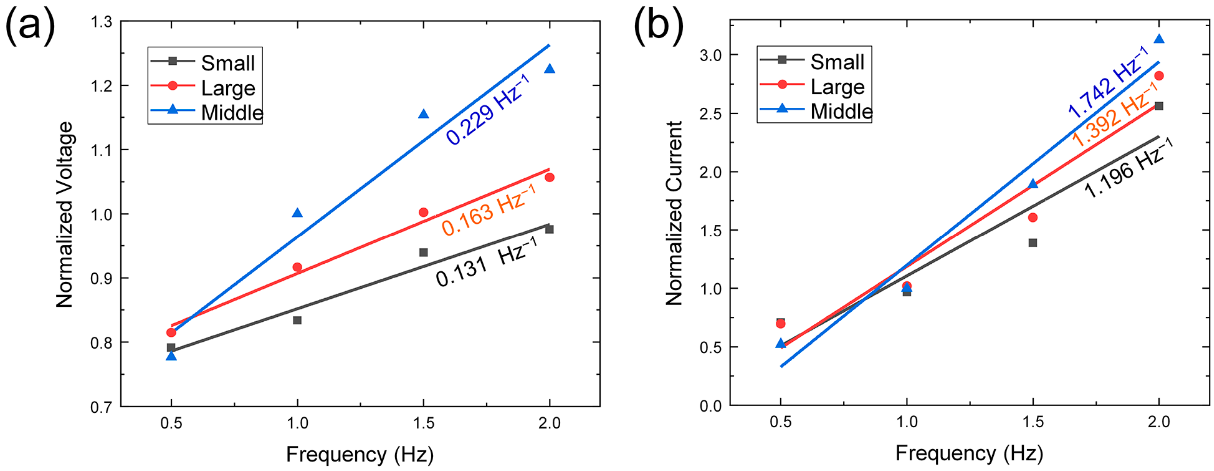

Figure S10. Normalized (a) voltage and (b) current with changing the frequency and device curvature of the DR-TENG.

11. Output comparison with and without the hydrophilic treatment-cases

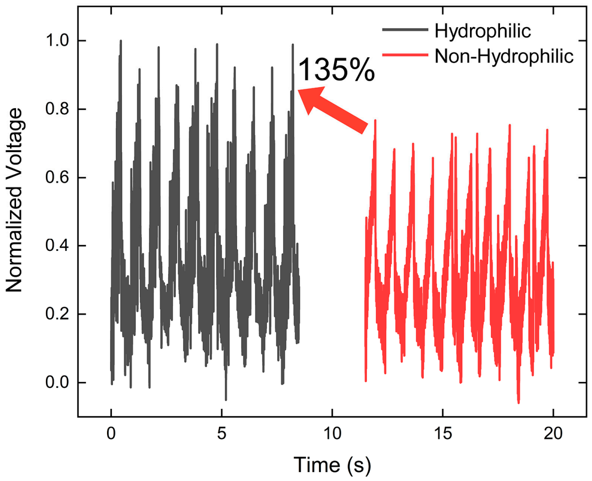

Figure S11. Normalized output voltage from the two surface conditions with and without acetone treatment at the outer ABS of the DR-TENG in the water wave condition.

12. Result of durability test in the water wave condition

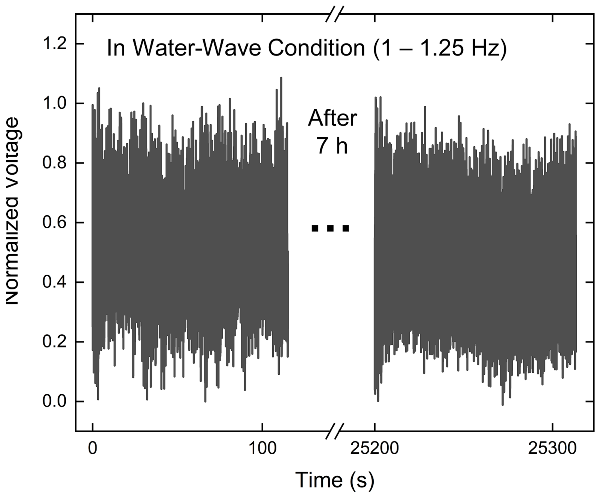

Figure S12. Normalized output voltage from the continuously operating DR-TENG in the water wave-condition for more than 7 hours.

13. Video of illuminating LEDs with DR-TENG

Video S1. Simultaneously illuminating five serially connected green LEDs with double rollers-case.

14. Video of water wave harvesting test in a water bath

Video S2. Water wave energy harvesting with locating the DR-TENG in a water bath.

15. Video of water wave harvesting test in the actual water wave condition with an anchor and operating a LED

Video S3. Water wave energy harvesting with the DR-TENG and an anchor in the actual water wave condition and operating a LED in real time.

16. Video of water wave harvesting test with array structure in the water wave condition

Video S4. Water wave energy harvesting with the 2 × 2 array structure of the DR-TENG in the water wave condition.
